# Patients with alcohol use disorder clustering based on definition of alcohol withdrawal syndrome

**DOI:** 10.1192/j.eurpsy.2025.955

**Published:** 2025-08-26

**Authors:** V. Karpyak, A. M.-C. Ho, B. J. Coombes, V. M. Pazdernik, J. A. Frank, J. M. Biernacka

**Affiliations:** 1 Psychiatry and Psychology; 2 Computational biology, Mayo Clinic, Rochester, United States

## Abstract

**Introduction:**

Alcohol withdrawal syndrome (AWS) is among the most severe components of alcohol dependence (AD). Severe AWS, especially complicated with seizures and delirium, was a common reason for medical complications and death. The introduction of benzodiazepines decreased but did not eliminate those risks. The definition of AWS evolved in different versions of Diagnostic and Statistical Manual for Mental Disorders (DSM) from having hand tremor plus one or more other symptoms in DSM-III-R to any two or more symptoms in DSM-IV, which was retained in the current version of DSM-5.

**Objectives:**

Comparing subgroups of individuals with AUD based on AWS symptoms profile to define phenotypes for investigation of biological underpinnings of AWS phenomenology and treatment response.

**Methods:**

Treatment-seeking individuals diagnosed with DSM-IV alcohol dependence (AD; n=473; 35.5% females) were assessed with Psychiatric Research Interview for Substance and Mental Disorders (PRISM), Timeline Follow back (past 90 days) alcohol consumption, Penn Alcohol Craving Scale (PACS), Pittsburgh Sleep Quality Index (PSQI), Patient Health Questionnaire-9 (PHQ9), and General Anxiety Disorder-7 (GAD-7). Latent class analysis was used to classify subjects according to lifetime prevalence of AWS symptoms according to DSM-III-R or DSM-IV/5. Demographic and other clinical variables were compared among clusters by linear model ANOVA and chi-squared test.

**Results:**

Four clusters were identified: subjects with (1) no history of meeting any AWS criteria (n=50); (4) with complete number of AWS symptoms, meeting both DSM definitions (n=259); and two clusters of subjects with smaller number of AWS symptoms: (3) those meeting only DSM-IV/5 criteria (n=94) and (2) those also meeting DSM-III-R criteria (n=70). Compared to cluster 2, the Cluster 3 members had higher frequency of anxiety, insomnia, and restlessness during withdrawal. The clusters also differed in the total number of drinks (p=0.021), average drinks per drinking days (p=0.013), history of anxiety disorder (p=0.006), substance-induced depression (p=0.019), and higher scores of PSQI (p<0.001), PHQ9 (p<0.001), and GAD-7 (p<0.001) with a relative increase in frequency between clusters as follows 1<2<3<4. Group 4 also had the highest percentages of subjects with history of seizure, delirium or hallucinations.

**Conclusions:**

Different definitions of AWS capture clusters of AD patients with different symptomatology, comorbidity and consumption patterns. Understanding of biological underpinnings behind those differences may guide improvement in personalized treatment selection.

**Disclosure of Interest:**

None Declared

